# The effect of a cold beverage during an exercise session combining both strength and energy systems development training on core temperature and markers of performance

**DOI:** 10.1186/1550-2783-9-44

**Published:** 2012-09-19

**Authors:** Danielle LaFata, Amanda Carlson-Phillips, Stacy T Sims, Elizabeth M Russell

**Affiliations:** 1 Director, Performance Nutrition, Athletes’ Performance, 2629 E Rose Garden Lane, Phoenix, AZ, 85050, USA; 2 VP Nutrition & Research, Athletes’ Performance & Core Performance, 2629 E Rose, Garden Lane, Phoenix, AZ, 85050, USA; 3 Research Associate ,Stanford Prevention Research Center, Stanford University School, of Medicine, Office Building 1265 Welch Road, Mail Code 5411, Stanford, CA, 94305-5411, USA; 4 Research Associate, Andrews Research and Education Institute, 1020 Gulf Breeze, Parkway, Gulf Breeze, FL, 32563, USA

**Keywords:** Hydration, Dehydration, Euhydration, Core temperature, Thermoregulation, Performance, Cold water, Exercise

## Abstract

**Background:**

Although studies have investigated the effects of hydration on performance measures, few studies have investigated how the temperature of the ingested liquid affects performance and core temperature during an exercise session. The hypothesis of the present study was that cold water would improve thermoregulation and performance as measured by bench repetitions to fatigue, broad jump for force and power and total time to exhaustion for cardiovascular fitness

**Methods:**

Forty-five, physically fit, adult males (30.28 ± 5.4 yr, 1.77 ± 7.8 m, 83.46 ± 11.5 kg; 13.7 ± 4.8 %BF; 49.8 ± 6.3 ml/kg/min V02) completed two 60-minute exercise sessions. Subjects consumed either COLD (4°C) or room temperature (RT) water (22°C) in randomized order. Core temperature was measured every 15 minutes throughout each trial using a digestible thermometer. Three performance tests were performed upon completion of the exercise session: bench press to fatigue, standing broad jump, and bicycle time to exhaustion

**Results:**

Although both groups significantly increased their core temperature (p<0.001) over the course of the exercise session and presented a significant decline in hydration status (p<0.001), participants in the COLD water trial had a significantly (p=0.024) smaller rise in core temperature (0.83°) over the duration of the trial in comparison to RT (1.13°). The participants in the COLD water trial were able to delay their increase in core body temperature for at least 30 minutes, whereas participants in the RT trial increased body temperature from baseline after 15 minutes. There was no significant difference between the COLD or the RT trials in broad jump and TTE performance tests. Bench press showed a small, albeit significant (p=0.046), decrease in performance when drinking COLD

**Conclusion:**

Drinking cold water can significantly mediate and delay the increase in core body temperature during an exercise session in a moderate climate with euhydrated subjects. The ingestion of COLD improved performance for 49% and 51% of the participants in the broad jump and TTE performance tests respectively, but did not reach statistical significance. Moreover, although minimal, subjects experienced a decrease in performance on the bench press during the COLD.

## Background

It is well known that exercising in hot environments can increase core temperature especially if dehydrated [1-4]. Dehydration impairs thermoregulation as well as cardiovascular, metabolic and central nervous system functions. Elevated core temperature has been reported to affect cognitive ability, elevate sympathetic nervous system activity, increase central fatigue, and ultimately lead to heat exhaustion/stroke if left unattended [5]. When considering that prolonged exercise in the heat has been shown to primarily be limited by thermoregulatory and fluid balance factors, it can be said that these physiological strains can negatively impact one’s ability to perform intense physical work [6]. Therefore, maintenance of a normal body temperature of 37°C, and hydration status during training is important for the success of an individual participating in physical activity. As a result, previous research has investigated the impact of water temperature on performance measures as well as core temperature regulation to determine the ideal fluid choice for optimal exercise performance. Currently, four studies have shown that there is a beneficial influence from beverage temperature on endurance exercise performance [2,3,7,8]. However, different exercise protocols and environmental conditions were used. Of the four studies, two reported large and significant improvement of endurance exercise performance (13% vs. 22%, respectively) in hot and humid conditions [2,3]. In contrast to these two studies, other investigations have reported that ingesting cold beverages during exercise in a cool to moderate environment does not improve endurance performance [7,9].

There is conflicting research on the impact of cold water consumption on thermoregulation. While some studies have failed to find a correlation between cold water consumption and decreases in core temperature, others have shown a link [2,8,9]. Reasons for this discrepancy include: (1) the fluid ingestion protocols differed greatly across all studies such that some required ad libitum vs. standardized at a bolus amount (900 ml before exercise and 100 ml every 10 minutes during); (2) The low exercise intensity protocol used in some of the studies may not have produced enough heat load to raise core body temperature to the level required to achieve a statistically significant difference between the treatment groups; (3) environmental conditions varied across all studies from 25°C to 40°C. It is important to note, studies conveying a decrease in core temperature through cold beverage consumption were conducted in hot and/or humid environments, and included the consumption of large intermittent bolus’ of cold water [3,5,10].

Due to the presence of conflicting research on cold water consumption’s impact on thermoregulation, the limited amount of studies investigating the influence of cold water consumption on exercise performance (especially strength and power measures) and limited general population data, it can be argued that more research on these topics is needed to determine the ideal hydration choice for the average general population exerciser.

It is the intent of the authors to investigate the effects of COLD (4°C) in comparison to room temperature (RT) water consumption (22°C) in physically fit males during a total body muscular strength and cardiovascular exercise session. To date, there is no literature investigating these effects in this population on this type of physical activity.

## Methods

### Subjects and screening

Subjects were recruited through a recruitment email and word of mouth to family and friends. Interested subjects that passed the screening questions were asked to attend a consent meeting. At this meeting, interested subjects learned about the study and had the opportunity to sign the consent form or decline involvement. Members of the research team facilitated the consent process. Each member of the research team had training in the protection of human subjects. They also signed a HIPAA form at this meeting and were given a copy of both the consent and the HIPAA for their records. All applicable institutional and governmental regulations concerning the ethical use of human volunteers were followed during this research.

All participants reported exercising at least five times per week with at least a six-week history of strength training three times per week. Participants were excluded for any of the following: known cardiac disease, uncontrolled hypertension, uncontrolled thyroid disease, uncontrolled diabetes, taking medications that could impair exercise performance (beta blockers), medical contraindications to exercise, an injury that prevented them from being able to complete movements in an exercise program, a doctor told them they cannot exercise or a VO2 below 35 mL/kg/min. Fifty-two healthy, physically fit males volunteered for the study. Data of seven subjects had to be removed as they started at least one exercise session in a dehydrated state. Therefore, 45 participants completed the trial (30.28 ± 5.4 yr, 1.77 ± 7.8 m, 83.46 ± 11.5 kg; 13.7 ± 4.8%BF; 49.8 ± 6.3 ml/kg/min V02) (Table 1).

**Table 1:** Summary of participant characteristics

**Variable**	
Age	30.28 + 5.4
**Anthropometric characteristics**	
Height (m)	1.77±7.8
Mass (kg)	83.46±1.5
**Body Composition**	
Body fat %	13.7±4.8
**Fitness**	
Estimated Peak VO^2^ (ml/kg/min)	49.69±6.3

Values represent mean ± standard deviation.

The study was approved by Compass Institutional Review Board (Mesa, Arizona) and written informed consent was obtained from each participant before enrollment.

### Experimental design

The study was conducted in a cross-over, randomized design. The null hypothesis that cold water will not impact core temperature or performance measures was tested via a repeated measures analysis of variance and the criterion for significance for all tests was set at p < 0.05. Participants undertook two experimental trials that were administered in simple blocks, randomized, crossover order, followed by three performance tests: (1) 60% 1RM bench press to fatigue, (2) broad jump, and (3) time to exhaustion (TTE) on a stationary Keiser bike. As participant blinding to drink temperature is impossible, the subjects were informed that that the study outcome of interest was body temperature not performance. Participants, in randomized fashion, were given either cold (COLD) 4°C, or room temperature (RT) 22°C water provided in vacuum insulated hydration bottles with Thermos® technology during the exercise protocols and were given 12 ml/kg in equal aliquots to be consumed during the rest period between sets of exercises and to be completed before the end of the exercise session and before the first performance test. In the subsequent exercise session the participants were given the exact amount of water they consumed during the first trial. The trials were separated by a minimum of four days and no more than 21 days. Participants were asked to refrain from strenuous activity and abstain from alcohol and caffeine consumption 48 hours prior to both exercise sessions. Participants were then asked to consume 8 ml/kg body weight of water to ensure euhydration starting at 3 hours priors to training session and to be finished ~45 min before arriving to facility. Each trial commenced at the same time each day to control for the effect of circadian rhythm on body temperature. The day of the exercise session, the participants were asked to ingest a biodegradable temperature sensor pill (CoreTemp capsule, Mini Mitter Co. Inc., Bend, Oregon, USA), with a small meal, 6–8 hours prior to the exercise session to allow adequate time for motility into the small intestine and to minimize the effects of swallowing cold liquids on temperature readings. Core temperature was monitored using a VitalSense telemetric physiological monitoring system (Mini Mitter Co. Inc., Bend, Oregon, USA). To control for the effect of diet and hydration on exercise performance the participants were asked to arrive at the training facility 1.5 hours prior to their scheduled exercise sessions to receive a standardized meal of 1.0 g carbohydrate/kg body weight and 0.4 g protein per kg lean body mass in the form of a shake to be finished within Â½hour prior to commencing the exercise session. Upon arrival to the training facility, 1.5 before commencing exercise session, the participants were asked to provide a urine sample cup for urine specific gravity analysis (USG) using Roche USG 10 urine strips. If a participant was dehydrated they were instructed to continue the 8 ml/kg body fluid protocol and re-test 45 minutes later to confirm they were hydrated. Core temperature was taken at baseline and every 15 minutes with the VitalSense telemetric physiologic monitoring system (Mini Mitter Co. Inc., Bend, Oregon, USA). Body weight and USG were taken prior to the exercise session and immediately after performing the TTE test.

During both trials, each participant was assigned an identification number which was placed on their own vacuum insulated individual Thermos® brand bottle. They were instructed to only drink from their own Thermos® brand bottle. During the cold trial, the drinks were cooled using a domestic refrigerator and maintained at 4°C. During the RT trial, drinks were maintained at 22°C. Temperature of the water was measured using a standard long glass mercury thermometer (Indigo® Instruments, Waterloo, ON, Canada). After the initial exercise session was completed, participants were given a five minute rest before commencing the performance tests. The performance tests were administered by the same person/investigator to avoid potential bias when providing encouragement. The performance tests included: flat bench to fatigue at 60% of one rep max (RM) to determine muscular endurance [11], broad jump to determine force and power production [12] and time to exhaustion (TTE) on stationary bicycle to determine cardiovascular endurance. For the broad jump test the subjects were asked to jump as far as they could horizontally on a flat surface 2 times. Both jumps were averaged. The endurance test (TTE) was administered using a modified McArdle (1973) bike protocol. The protocol was based on the use of the Keiser stationary bike. The watts are based on the gear and the participants had to hold 80 rpms at each gear. The ramping was adjusted to fit the gearing designed of the Keiser stationary bike. We used it as a sub max test based on maintaining 80 rpms. If the participants could not keep above 80 rpm then the participant was instructed to stop and gear, time and Core temperature were recorded.

### Preliminary measurements

Subjects completed the baseline testing at least four days prior to their first testing day. After the completion of the baseline testing, subjects were briefed on the study design and the drinking and exercise protocol. They were also able to familiarize themselves with the performance tests that they were to perform at the end of their exercise sessions. On their first trip to the facility, the participants’ weight, height, and 7-site skin fold thickness were measured. Skin fold thickness measurements were taken at seven sites (triceps, subscapula, chest, mid-axillary, abdominal, iliac create, front thigh) using calipers (Lange Skin fold Caliper, Beta Technology, Santa Cruz, CA). Percent body fat was determined using the Siri equation and body density was calculated with the Jackson-Pollock equation. After anthropometrics were taken participants proceeded to the flat bench press to determine the bench press 1RM performance test. Subjects were asked to bench press 60% of their 1RM as many times as they could. During the test subjects had a spotter behind them to take the weight once the subject fatigued. The participants were also fitted and assigned a stationary bike for the time to exhaustion performance test. Lastly, estimated peak oxygen consumption was assessed to determine fitness levels using a treadmill (Woodway, Waukesha, WI) via an 8–12 minute ramping protocol during which the American College of Sports Medicine graded walking equation was applied (American College of Sports Medicine, 2010). During the submaximal protocol, heart rate and ventilation were measured using the iMett system (Woodway, Waukesha, WI). Ventilation was measured with a flow meter and mask (Hans Rudolph) from which a ventilatory threshold was determined. Adjusted ACSM max norms to 95%, as a submax test was administered. A VO2 of ≥35 ml/kg/min was considered moderately fit and approved to participate in the study.

### Exercise protocol

Subjects partook in an exercise session that combined both strength and aerobic exercise. The exercise session was supervised and led by a Performance Specialist at Athletes’ Performance. The subject to trainer ratio did not exceed 10:1. The 60 minute exercise session consisted of a 5 minute warm-up with dynamic stretching, 5 minutes of medicine ball exercises, 35 minutes of full body strength training and 15 minutes conditioning. The strength sessions exercises consisted of four exercise blocks which included a strength/power exercise followed by a corrective exercise/stretch to facilitate active rest between sets. There was a prescribed 2–3 minute rest between strength blocks 1 and 2, and 1–2 minutes rest between strength blocks 2 and 3, and 3 and 4. The strength session consisted of: 1st block (2x6):Dumbbell squat to press, power/velocity emphasis, loading was approximately 55% of 1RM, ½ kneeling quad hip flexor stretch for 30s-1m rest in between sets. Second block (3x10): Dumbbell flat bench press (strength/hypertrophy emphasis), loading was approximately 75% of 1RM with bent over T’s (3x6) during 30s-1m rest in between sets; Double leg, dumbbell Romanian deadlift (strength/hypertrophy emphasis), loading was approximately 75% of 1RM with bent knee hamstring stretch (3x6) during 30s-1m rest in between sets. Third block (2x8): 1 arm rotational row (power/velocity emphasis, loading was approximately 55% of 1RM with a push up position hold with alternating arm lifts during 30s-1m rest period. Fourth block (2x8): Lunge to curl to press (strength/hypertrophy emphasis) loading was approximately 80% of 1RM, with a front plank/pillar hold (1 minute) during rest in between sets; Eccentric only slide board leg curls. The 15 minute conditioning/cardiovascular exercise that followed the strength training was designed as high intensity interval training. Intensity was determined based on each participant’s individual heart rate zones which were prescribed off their sub max treadmill VO2 test results as 65-85% of ventillatory threshold (VT), 100-110% of VT, and 110% VT-Peak HR. The cardio session started with a 3 minute warm up (65%-85% VT) with goal of heart rate being in this zone by the end of the 3 minutes. Two minutes was spent at 100-110% VT, 1 minute at 100%VT-Peak, 1 minute at 65-85% VT, 2 minutes 100-110% VT, 1 minute 100% VT-Peak, 1 minute 65-85% VT, 2 minutes 100-110% VT, 1 minute 100% VT-Peak and 1 minute 65-85% VT.

During the exercise session, subjects were asked to drink their assigned beverage during rest periods between exercise sets. Consumption of water during the exercise session was ad libitum and the participants were instructed to completely finish the water by the end of the exercise session. If water was left-over it was recorded (only four participants did not consume all water). Core temperatures were measured every 15 minutes during the session via the VitalSense telemetric physiologic monitoring system (Mini Mitter Co. Inc., Bend, Oregon, USA) the researcher held near the subject’s body.

### Environmental conditions

Wet and dry bulb temperatures were taken during each trial. Wet bulb temp averaged 14.9°C and 15°C (p=0.6273) for both RT and COLD trials respectively and dry bulb temp averaged 24°C and 24.2°C (p=0.1179).

### Statistics

A statistical analysis was performed by the authors. Data were ensemble averaged across all 45 participants and standard deviations were calculated. The study design was a randomized cross-over study. Paired t-tests were used to compare performance between conditions and to compare the absolute change in body temperature from the pre-exercise session to the post-exercise session. A repeated measures analysis of variance was used to test for a significant effect of group, time and the interaction between the two during the hour of exercise. Tukeys post-hoc tests were used to determine significant differences between time points. Criterion for statistical significance was set at p<0.05.

## Results

Body temperature in the COLD condition changed 2% from baseline to post-exercise session (37.06 ± 0.72°C to 37.79 ± 1.16°C). Body temperature from baseline to post-exercise session changed 3% in the RT condition (36.85 ± 0.98°C to 37.94 ± 0.82°C). Although both groups significantly increased their core temperature over the course of the training and testing session (p<0.001), participants in the COLD water trial had a significantly (p=0.024) smaller rise in core temperature (0.83°± 0.63°) over the duration of the trial in comparison to RT (1.13° ± 0.78°) Table 2.

**Table 2:** Core temperature over duration of the trial

	**Core temperature (°C)**					
	**Baseline**	**15 min**	**30 min**	**45 min**	**60 min**	**Post performance tests**
COLD	37.06±0.72	37.19±1.09	37.38±1.25	37.55±1.17	37.79±1.16	37.89±0.65
RT	36.85±0.98	37.23±0.96	37.45±1.05	37.55±1.17	37.94±0.82	37.98±0.51

**Figure 1:**
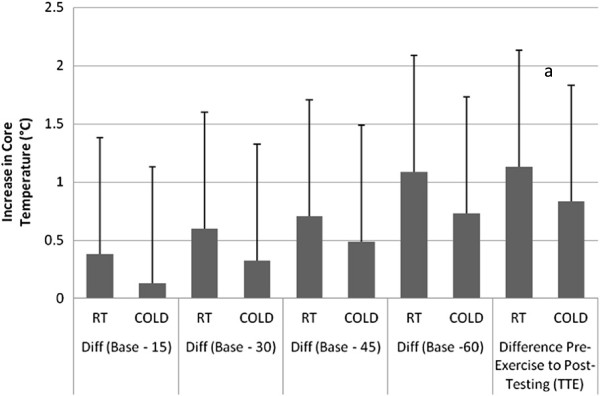
**Comparison of core temperature increase over the duration of the trial.**^a^p<0.05.

There was a significant effect for time such that body temperature increased in both groups over the course of the 60-minute exercise session (p<0.001). There were no significant interactions between condition and time (p=0.380) such that subjects behaved similarly to the effect of exercise over time, regardless of water temperature condition. The post-hoc analysis of changes in body temperature over time indicates that, when drinking RT water, a significant increase in body temperature was observed after 15 minutes. In the COLD condition, the increase in body temperature was delayed until 45 minutes. There were no significant interactions between condition and time (p=0.141) such that subjects behaved similarly to the effect of exercise over time, regardless of water temperature condition. Figure 1 shows the change in core temperature from baseline at each 15-minute time point.

There were no significant differences between the groups (during the RT condition and COLD condition) in body mass (p=0.919). There was, however, a significant effect of time (p<0.001) such that body mass significantly decreased from the pre-exercise to post-exercise time point in both groups. In the COLD condition body mass decreased from 85.2 ± 11.6 kg to 84.8 ± 11.3 kg and in the RT condition body mass decreased from 85.2 ± 11.6 kg to 84.7 ± 11.3 kg. There were no significant interactions (p=0.223) and subjects did not behave differently to the conditions at the time points.

Additionally, there were no significant differences in hydration status between the COLD and RT conditions at either the baseline time point (p=0.549) or the post-exercise time point (p=0.368). Since atmospheric conditions were also held constant, the authors can infer that water intake was not different between COLD and RT conditions. Both groups experienced a significant decrease in hydration status from beginning of the exercise session to post-exercise session in both conditions (p<0.0001). There were also no significant interactions between groups and the pre to post-training time point (p=0.209) Table 3.

**Table 3:** Hydration status during training

	**COLD**		**RT**	
**Test**	**PRE**^a^	**POST**^a^	**PRE**^a^	**POST**^a^
Weight (kg)	85.2±11.6	84.8±11.3	85.2±11.6	84.7±11.3
Hydration Status (urine specific gravity)	1.00615±0.005	1.01021±0.005 ^b^	1.00564±0.004	1.011942±0.013^b^

^a^Values represent mean ± standard deviation.

^b^p < .05. Criterion for statistical significance was set at p<0.05.

When investigating measures of performance, there were no significant differences between COLD and RT groups for two of the three performance tests: broad jump (p=0.465) or TTE (p=0.735). Subjects performed broad jumps of 2.17 ± 0.27 m and 2.15 ± 0.25 m in the COLD and RT conditions, respectively. TTE was 638 ± 187 seconds and 643 ± 189 seconds for the COLD and RT conditions, respectively. However, even though there was not a significant improvement demonstrated, 49% of the participants improved in the broad jump and 51% in the TTE respectively during the COLD. Subjects participating in the RT condition were able to perform significantly more bench press repetitions to failure than when they participated in the COLD condition (p=0.046). During the COLD condition 22 ± 3.5 repetitions (range: 15–30) were performed and during the RT condition 22.7 ± 3.2 repetitions (range: 17–31) were performed. This was a small, albeit significant, improvement; however, a calculation of an effect size [11] indicates that this would be a negligible to small effect (d=0.2) Table 4. 

**Table 4:** Summary of performance test results

**Test**	**COLD**^**a**^	**RT**^**a**^	**% of subjects who had improved performance during COLD**
Bench (reps)	22 ± 3.5	22.73 ± 3.2^b^	14%
Broad Jump (m)	2.17 ± 0.27	2.15 ± 0.25	49%
TTE (seconds)	638 ± 187	643 ± 189	51%

^a^Values represent mean ± standard deviation.

^b^p< 0.05. Criterion for statistical significance was set at p<0.05.

## Discussion

Current literature reports that a rise in core temperature can significantly impede performance [1]. Endurance exercise capacity has specifically been found to be affected by a hot environment, which leads to a rise in core temperature and increased dehydration [13-15]. Burdon et al., found that consuming cold beverages according to the ACSM guidelines, in euhydrated subjects, enhanced endurance performance in a hot environment [1]. In this study subjects consumed, at each separate trial, a sports drink at the following temperatures and times: 37°C and 4°C consumed every 10 minutes (2.3 mL/kg) and 30 mL ice puree (−1.0°C) every 5 minutes with holding it in the mouth for at least 30 seconds before swallowing during the 90 minute exercise session. Even though this study concluded that there was an improvement in exercise performance with the cold beverage and ice puree, this study has a confounding factor in that it used a sports drink instead of plain water. One could hypothesize that the extra fuel (carbohydrate) and electrolytes acted as ergogenic aids and combined with being cold or alone enhanced performance.

Most studies have addressed a rise in core temperature with a dehydrated population during hot and/or humid conditions over a longer period of time [7,8]. It is important for the elite and physically fit individuals alike to maintain a normal body temperature (37°C). Some literature suggests that consuming large amounts of cold fluid during exercise would allow the body to have increased capacity to store heat (i.e. heat sink), thereby reducing heat gain during exercise.

Seven studies have investigated the effect of beverage temperature on core body temperature during exercise [2,3,6-10], however, the methodologies and protocols vary widely. Four of the seven studies concluded that consuming a cold beverage during exercise resulted in a lower core temperature at the end of the exercise session compared to consuming a warm beverage. Our study was unique in that at the time the trial started there had not been a published paper on the effects of COLD vs. RT water during a traditional exercise session (60 minutes) in physically fit individuals, in a moderate climate. No studies have investigated the effect of cold water on thermoregulation and a traditional exercise session combining both strength and endurance training in physically fit individuals.

In our study we found that while ingesting the COLD water, subjects were able to significantly mediate their rise in core temperature over the entire duration of the study (ie, when comparing the magnitude of the change in core temperature, subjects who drank COLD water had a significantly lower change in core body temperature than subjects who drank RT water (p=0.024)). Subjects finished their water allotment at the end of the exercise session before commencing the performance tests and the core temperature mediation continued in the COLD trial through the end of the performance tests (p=0.024). Although there was not a statistically significant improvement in the broad jump or TTE performance tests while drinking the cold water, approximately 50% of the subjects performed better during the COLD trial in both tests. As for the bench press performance test, even though participants were randomized, of the 45 participants, 26 performed the bench press under the RT condition on their second exercise session. The significantly better results during the RT may have been skewed due to the fact that this was their second time performing this type of test during the RT condition and may have known more about what to expect and were motivated to improve their reps to fatigue from their previous test. Another possible explanation for the bench press results is that the calculated effect size was low. However, for both athletes and physically fit individuals, the ability to train longer and harder is important. For athletes, a few seconds can mean the difference between first and second and one last burst of power can mean scoring the winning points. Therefore, the improvements for the subjects are relevant to their environments.

The temperature of the COLD water trial was chosen to be representative of water stored in a general household refrigerator and RT was chosen to be representative of the room temperature. We found that the COLD water trial resulted in significantly less of a change in body temperature from pre-exercise session to post-performance testing after a 60 minute exercise (p=0.024). The change was 1.1°C (±0.8) in the RT condition and 0.8° (±0.6) in the COLD condition; therefore, we have found that ingestion of a cold beverage significantly improves the body’s ability to maintain core temperature. These findings are similar to that of Armstrong et al., Lee et al. and Szlyk et al. [6,9,10], however, these studies were conducted in the heat at 40°C, 35°C and 40°C, respectively.

Although there was not a significant benefit of COLD water in the performance tests measured, the COLD water clearly helped the participants to maintain core body temperature during exercises, which may have other positive impacts. Current literature also reports that a rise in core temperature can significantly impede performance [1].

There is debate as to the core temperature threshold where a decrease in performance starts to occur. Core temperatures at fatigue have been reported to be between 38.4°C and 40°C [2,16]; however, many studies report that exhaustion occurs well below 40°C and that the variability may be due to training status, body composition, or various core temperature collection methods [2]. Burdon et al., evaluated performance during a 90 minute steady state exercise session in the heat and reported final rectal temperatures of 38.3°C for their COLD group and 38.5°C for their thermoneutral group [1]. In our study, the maximum core temperature readings were at 37.98°C ± .51 and 37.89°C ± .64 for the RT and COLD groups respectively, which are lower than studies done in the heat and below previously reported thresholds for fatigue. Although core temperature was significantly different from pre-exercise session to post-testing, a possible reason why we did not find significant improvements in performance measures may be due to the fact that the participant’s core temperatures did not reach a high enough temperature for the COLD water to create a performance impact. Therefore, the impact of COLD on performance measures may be more evident at higher temperatures.

Most studies have addressed rise in core temperature with a dehydrated population during hot and/or humid conditions over a longer period of time [6,7]. Future research may address the effects of a cold water trial in a 90–120 minute exercise session on rise in core temperature. Even though there was not a significant improvement for subjects when drinking COLD water prior to performance tests, overall performance measures may not be sensitive enough to measure the small changes that COLD water may have. Moreover, if the same study was done with dehydrated subjects or in a hot/humid environment, there may have been a greater performance benefit exhibited.

The repeated measures analysis of variance showed no significant interactions (p=0.286), indicating that subjects did not perform significantly different over time in one condition than in the other. There was also no significant effect of group (p=0.619). There was, however, a significant effect of time (p<0.001).

There were two limitations to this study. Environmental conditions of temperature and humidity were controlled throughout the study at a constant value. Secondly, the total duration of the study was less than 90 minutes. COLD water may provide the most benefits in stressful environmental conditions (higher temperatures and humidity levels and/or longer duration of exercise) [1], but the current study did not test these independent variables.

## Conclusion

This study found that drinking COLD water during a traditional exercise program in a moderate climate can have a significant impact on the body’s ability to maintain core temperature. The benefits for reducing the rise in core temperature did not translate to significant improvements in power, aerobic endurance, and muscular endurance-based exercises. Secondary to the significant impact of the COLD water on the body’s ability to maintain core temperature, it is still recommended that both, athletes and physically fit individuals, consume COLD beverages during exercise. Delaying a rise in core temp may have positive impact on exercises not investigated in this study, but it’s unlikely to have negative effects. It is recommended that further work be done to further investigate the impact of COLD water consumption on strength and power performance in an extreme environment, with dehydrated subjects, or specific exercise bouts of longer duration.

## Competing interests

This study was funded by Thermos L.L.C. (Schaumburg, IL, USA).

## Authors’ contributions

DL was the study coordinator and was involved in research design, data collection and analysis, as well as manuscript preparation. ACP and was involved in research design, analysis and manuscript preparation. SS assisted in research design. ER assisted in data analysis and manuscript development. All authors read and approved the final manuscript.
